# Zinc Deficiency in Men Over 50 and Its Implications in Prostate Disorders

**DOI:** 10.3389/fonc.2020.01293

**Published:** 2020-08-06

**Authors:** Ann Katrin Sauer, Hector Vela, Guillermo Vela, Peter Stark, Eduardo Barrera-Juarez, Andreas M. Grabrucker

**Affiliations:** ^1^Department of Biological Sciences, University of Limerick, Limerick, Ireland; ^2^Bernal Institute, University of Limerick, Limerick, Ireland; ^3^Health Research Institute (HRI), University of Limerick, Limerick, Ireland; ^4^Vela Staines y Asociados SA de CV, Monterrey, Mexico; ^5^Zinpro Corporation, Eden Prairie, MN, United States; ^6^Autismo ABP, Monterrey, Mexico; ^7^Tecnologico de Monterrey, Escuela de Medicina y Ciencias de la Salud, Monterrey, Mexico

**Keywords:** ZnAA, prostate cancer, benign prostate enlargement, Zn, prostatic hyperplasia, supplement

## Abstract

Research has been consistently showing the role of zinc (Zn) in prostate function. In this article, we review the current literature on the anatomy and main functions of the prostate, highlighting the role of zinc. In particular, we will review the etiology of benign prostate enlargement (BPH), its prevalence in men over 50, the likelihood of BPH becoming prostate cancer (PCa), and explain the relationship of zinc and apoptosis in the prostate cells and the implications for BPH and PCa. We present a model that explains how endogenous factors provoke excretion of zinc or limit zinc absorption, and how exogenous factors like nutrition and drugs regularly used in men over 50 can significantly decrease zinc status and thereby increase the risk of BPH. Finally, we explain how Zn amino acid (AA) complexes may be capable of avoiding antagonists and inhibitors of zinc absorption, thereby increasing the bioavailability of zinc for the necessary biological processes in the prostate.

## Introduction

### Anatomy and Function of the Prostate

**Figure 1:**
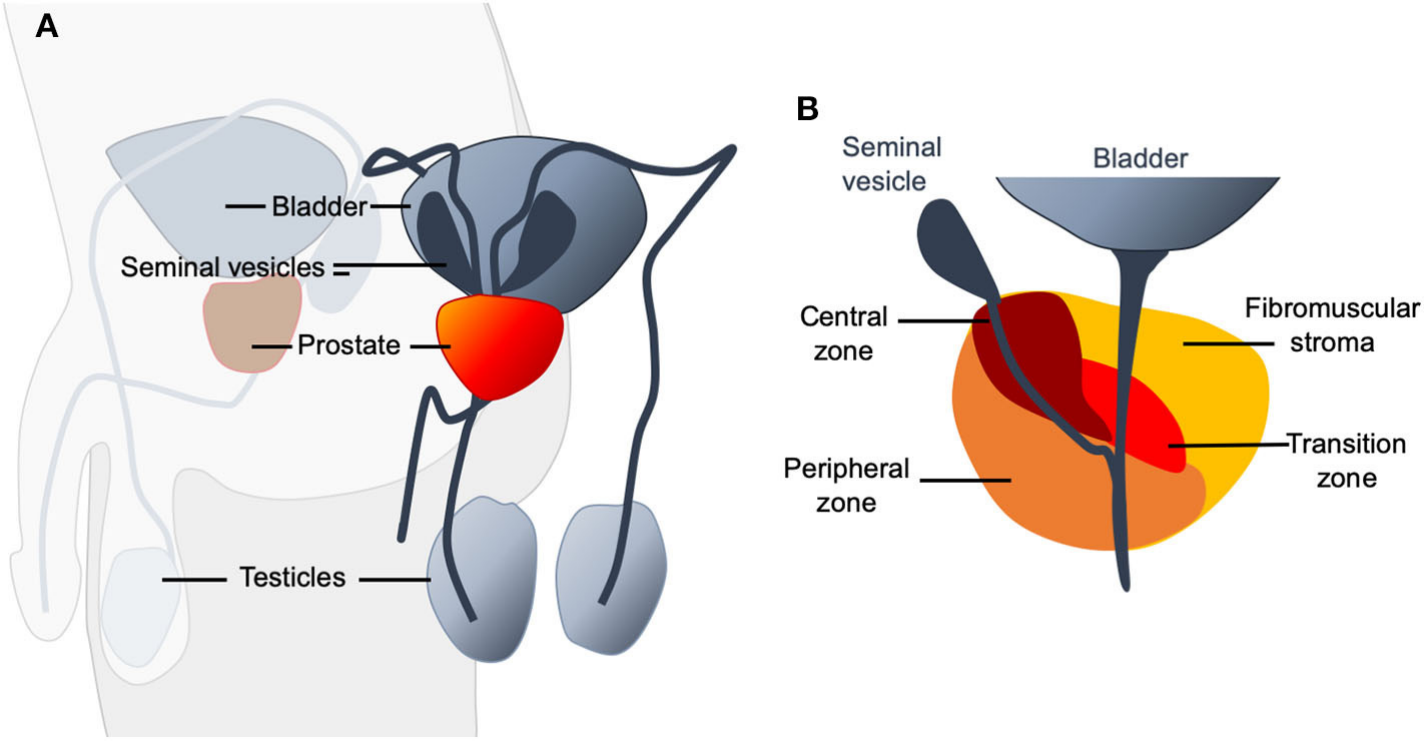
**(A)** Schematic representation of the location of the prostate (shown in red) in the male urogenital system. It is an organ of fibro-muscular and glandular nature and has the shape of an inverted pyramid. **(B)** Four zones can be distinguished in the prostate: The anterior zone or fibromuscular stroma, the peripheral zone, the central zone, and the transitional zone. The anterior zone is of fibromuscular nature, a thick sheet of compact connective and muscular tissue that covers the entire anterior surface of the prostate. It surrounds the proximal urethra at the level of the bladder neck, where it joins the internal sphincter and detrusor muscle in which it originates. It occupies almost a third of the total volume of the prostate and does not contain glands. It does not participate in any pathology of the prostate. The peripheral zone is of endodermal origin and is the largest anatomical region of the glandular prostate. The central zone is the smallest of the regions of the glandular prostate. It represents between 20 and 25% of its mass and is crossed by the ejaculatory ducts. The fourth zone, the transitional zone, has a mesodermal origin, formed by a small group of ducts closely related to the proximal urethra. These ducts represent 5% of the glandular prostate mass.

The prostate is a gland located in the man's pelvic cavity, behind the pubis. It is in front of the rectum and under the bladder. It wraps and surrounds the first segment of the urethra just below the bladder neck (Figure 1A).

The prostate is part of the urinary and reproductive system, anatomically related to other structures such as the vas deferens and seminal vesicles. The glandular tissue of the prostate is distributed in three histologically defined areas and immersed in several muscle layers, with little presence of connective tissue, which ultimately creates three lobes: two lateral and one medium.

The anatomical model that is currently accepted distinguishes four zones in the prostate (Figure 1B). The most critical areas with regards to prostate diseases are the central and peripheral zones of the prostate. Almost all prostate carcinomas originate in the peripheral zone (1). The anatomical characteristics favor that all the changes and pathological processes, both benign and malignant, that occur in this gland cause more or less notable alterations in urination (2).

The primary function of the prostate gland as a male accessory sex organ lies in the secretion of prostatic fluid, a component of semen that contributes between 15 and 30% of overall seminal fluid volume (3). Through its smooth muscle layer, the prostate ensures that the seminal fluid is pressed into the urethra during ejaculation (4). To prevent seminal fluid from reaching the bladder during ejaculation, the prostate muscles and the urethral sphincter contract effectively, closing off the urethra toward the bladder (3). The prostatic fluid contains various enzymes, citric acid, and high amounts of monovalent and divalent metal ions such as zinc (3, 4).

### Benign Prostate Hyperplasia (BPH)

Although the size of the prostate varies with age, in young and healthy men, the normal gland size is about 3 × 3 × 5 cm (25 ml volume) and it weighs between 15 and 20 g. Usually, this remains stable until men reach their 40's, the age in which a series of histological changes occurs: the gland grows and blocks the urethra or bladder, causing difficulty in urinating and interference in sexual functions that may eventually lead to benign prostatic hyperplasia (BPH) (2, 5). BPH is defined by histological alterations primarily within this prostatic transition zone, characterized by the proliferation of the epithelium and smooth muscle (6). According to McNeal (7), BPH develops in two phases. Within the first 20 years of BPH development, it is defined by an increase in the number of BPH nodules, while during the second phase, BPH is primarily characterized by an increase in the size of glandular nodules (7, 8). Problems for patients can arise in two ways, by direct bladder outlet obstruction (BOO) due to size of the enlarged prostate (static component) or by an increase in smooth muscle tone within the prostate (dynamic component), potentially manifesting in lower urinary tract symptoms (LUTS) (6, 9, 10). BPH is a common age-related phenomenon in men. While half of the men in their 60 s (50–60%) develop hyperplasia, by the time men reach the age of 70 and 80 years of age, 80–90% are affected (11, 12). Although not every man with BPH will necessarily be affected by significant LUTs, most common complaints include weak urine flow, straining, hesitancy, pro-longed voiding, complete or partial retention of urine, overflow incontinence of the bladder or irritative symptoms such as nocturia, painful urination and urge incontinence (12).

### Transformation of Normal Prostate to BPH: BPH as a Precursor of Prostate Cancer (PCa)

While the development of BPH is not yet completely understood, the etiology appears to be under endocrine control and multifactorial (13). Common consensus explains the volumetric increase of the prostate gland due to reduced apoptosis and cellular hyperplasia. This theory is defined as the “primordial cell” theory (14).

The development of BPH often begins around 40 years of age with a focal phenomenon of stromal origin. From the age of 50, there is then a global and rapid increase in volume due to an increase in the number of fibromuscular and glandular tissue cells, both in the periurethral and transitional areas. Thus, the increase in prostate volume is caused by excessive cell growth and a limited process of apoptosis, caused by the imbalance of growth-promoting factors and that of inhibitors, manifesting as pathology. Thereby, the leading causes of cell growth are:

Testosterone and Dihydrotestosterone (DHT): Within the prostate, the hormone testosterone is converted to DHT by 5-alpha reductase. Besides stimulating prostate growth and development, DHT seems to be instrumental in the progression of BPH pathology (9). While increased serum concentrations of testosterone itself seem not to increase the risk of developing BPH, several studies report that increased levels of DHT or DHT metabolites (17b-diol-glucuronide, androstanediol glucuronide) promote BPH (9, 15, 16). In line with these findings, medical intervention in the form of 5-alpha reductase inhibitors (finasteride and dutasteride) for treatment of BPH and resulting LUTS hinders the further progression of BPH, by decreasing DHT concentration in serum of patients (9, 17, 18).

#### Lifestyle

Besides endocrine factors such as DHT, lifestyle factors, especially those associated with metabolic abnormalities connected to cardiovascular disease, pose a risk for developing prostatic diseases such as BPH and prostate cancer. These factors lead to the so-called metabolic syndrome, which includes glucose intolerance, hypertension, and obesity (9, 19).

Prostate cancer (PCa), as the second most diagnosed type of cancer in men and BPH with a majority of men afflicted at an older age, show commonalities on the genetic, molecular, and cellular level. This suggests an association of BPH with prostate cancer development, if not causality (20). Due to the similarities in pathogenesis and the role of androgens in growth, antiandrogenic drugs, 5-alpha reductase inhibitors, and gonadotropin-releasing hormone agonists are useful for the treatment of both BPH and PCa (20–22). However, although BPH has been suggested to be a risk factor for PCa, which has been confirmed in a meta-analysis (23), the underlying mechanisms that may explain a causal link in the relationship between BPH and PCa are currently not well-known (24). In addition, another study proposed that BPH may actually slow down tumor formation by mechanically impeding tumor growth (25).

BOO and BPH, when associated with LUTS, impact public health considerably (9). A study reports 3.7 million emergency room visits within 3 years (2007 to 2010) by men in the US due to urinary retention (9, 26, 27). With an increasing incidence rate of BPH and associated complications, public health costs for diagnosis and treatment are substantial. Estimates of the annual cost for provided health care services within the US for BPH treatment range around $3.9 billion in 2014 and might be much higher to date (9).

## Discussion

### The Role of Zinc in Normal Prostate Function and Metabolism

**Figure 2:**
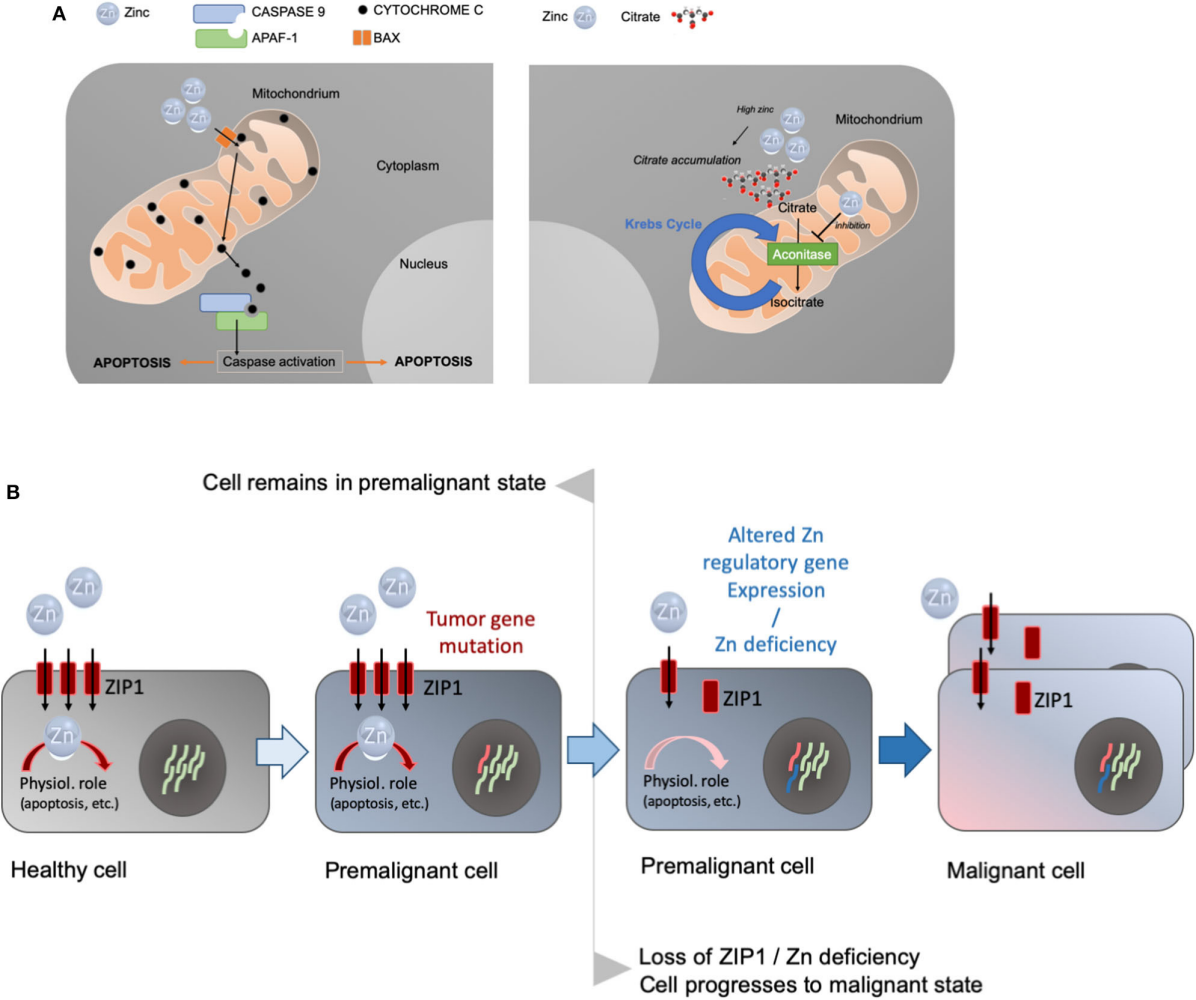
**(A)** Prostate cells accumulate high levels of zinc. Zinc enters the mitochondria causing cytochrome c to be released from the mitochondria, triggering caspase activation and the induction of apoptosis (left panel). Alternatively, zinc can inhibit aconitase, the enzyme catalyzing the production of isocitrate from citrate in the Krebs cycle (right panel). This leads to the accumulation of high levels of citrate that is characteristic for prostate cells. **(B)** The concept of the oncogenetic and genetic/metabolic transformations in the carcinogenesis process, and the role of zinc and zinc transporters in the etiology of prostate cancer. Premalignant cells may have predisposing mutations in tumor genes. A decrease in zinc and/or decrease in the ZIP1 transporter in prostate cancer occur as early events in premalignant cells and precede malignancy. Zinc deficiency and/or the downregulation of zinc import proteins subsequently results in a malignant cell.

To produce and secrete high levels of citrate, the prostate accumulates high levels of zinc in specialized acinar epithelial cells of the peripheral zone. Zinc levels and citrate metabolism are linked in the prostatic gland. Zinc inhibits mitochondrial aconitase (28). M-aconitase is responsible for the catalyzation of the oxidation of citrate to isocitrate as part of the first step in the Krebs cycle. The inhibitory effect of zinc results in the accumulation of citrate in the mitochondria before it is exported to the cytosol and secreted as a major component of prostatic fluid (29). Thus, the high levels of zinc in the prostate ensure the inhibition of M-aconitase while zinc that occurs outside the prostate in lower levels cannot act as a competitive inhibitor (29–31). The zinc transporter ZIP1 (Zrt- and Irt-like proteins 1, SLC39A1) is responsible for the uptake of zinc into prostate cells. The upregulation of ZIP expression in prostate cells ensures the accumulation of zinc in the prostate (29) (Figure 2A), a process that may be regulated by testosterone and prolactin (32).

Besides its role in the truncation of the Krebs cycle resulting in decreased energy production in the prostate and release of high levels of citrate in prostatic fluid, zinc accumulation in prostate cells can be an inducer of mitochondrial apoptosis, thereby effectively inhibiting proliferation and growth of the tissue (33). In *in vitro* studies, non-zinc-accumulating prostatic cells do not show apoptogenic properties, while zinc accumulating cells exhibit these capabilities suggesting that apoptogenesis is a unique feature of zinc accumulating prostate cells (33). Zinc hereby is taken up into the mitochondria of the prostate causing cytochrome c to be released from the mitochondria, triggering caspase activation and the induction of apoptosis (Figure 2A).

Intriguingly, both zinc and citrate are diminished in prostate cancer, implicating an essential role of high zinc levels for maintaining a non-malignant prostate.

### Zinc Deficiency in Prostate Disease

More than 16 studies have reported that zinc is markedly decreased (~60–80%) in prostate cancer compared to normal and benign prostate tissue and no study reported prostate cancer without decrease in zinc levels so far (32).

High levels of zinc are essential for maintaining prostate health and function due to its role in apoptosis and truncation of the Krebs cycle (citrate accumulation) (34). While this unique metabolic process in prostate cells ensures high levels of citrate release in the prostatic fluid as a major component in semen, it negatively affects the process of energy generation. Hence, when prostate cells undergo malignancy, and cancerous cells lose their capability to accumulate zinc, the continuation of the Krebs cycle releases energy, making malignant cell growth in the prostate more energy-efficient for the cells (35–37).

Taking this into consideration, insufficient zinc levels might have troublesome repercussions for men. Indeed, in prostate cancer tissue, mean zinc levels are decreased by up to 80%, and in prostatic tissue derived from BPH, zinc levels are decreased by more than 50% (38). In addition, a significant (on average, 44%) increase was observed in the urine zinc/creatinine levels in prostate carcinoma when compared to BPH, and a highly significant increase (on average, 53%) when compared with controls was found, hinting at increased zinc excretion. There was also already a significant increase in urine zinc excretion in men with BPH compared to men with a healthy prostate. This suggests that pathological conditions of the prostate gland in patients with BPH or carcinoma may be associated with an alteration in biochemical parameters such as a reduction in the level of tissue zinc, plasma zinc, and an increase in urinary zinc excretion (38). For example, a recent study found that PCa patients had markedly reduced plasma zinc levels and that a low zinc status was more pronounced within the severe grade and advanced PCa disease subgroups, suggesting that low zinc status is associated with PCa (39).

### Hypothesis: Low Zinc Status Is the Possible Cause of Both BPH and PCa

Pieces of evidence have accumulated that support this hypothesis: Costello and Franklin (37) proposed “a modified concept of the carcinogenesis process that incorporates a multistep oncogenetic transformation of normal cells to neoplastic cells, and genetic/metabolic transformation of the neoplastic cells to premalignant cells and ultimately to malignant cells” (Figure 2B).

In this concept, a key role of zinc in the transformation process was proposed. A decrease in zinc levels does not necessarily trigger malignancy without an initial oncogenetic transformation from the normal state to a neoplastic state with malignancy potential. However, a decrease in zinc levels and/or downregulation of ZIP1 will facilitate the transformation from a neoplastic cell with malignant potential to a malignant cell. This ZIP1/loss of zinc transformation occurs in almost all cases of prostate cancer. The downregulation of ZIP1 may be linked to abnormal REBB-1 (ras responsive binding element protein-1) function. REBB-1 is a transcription factor regulating ZIP1 gene expression and its activity is controlled by RAS. Unfortunately, RREB-1 regulation via RAS signaling remains poorly understood and the involved genes/signaling pathways await identification (40, 41).

Due to zinc's role as a stepping stone in developing malignant cells, restoration of normal zinc levels may be a promising approach in prostate cancer prevention and treatment (28). In addition, decreased levels of zinc may lead to an increased activation of the ZnR/GPR39 receptor in prostate cancer cells. ZnR/GPR39 is a plasma membrane G-protein coupled receptor that is sensitive to zinc. ZnR/GPR39 activation in prostate cancer cells was shown to promote cell growth through PI3K dependent upregulation of ERK and AKT phosphorylation (42). However, following ZnR/GPR39 desensitization, ERK phosphorylation was diminished in prostate cancer cells (43). ZnR/GPR39 desensitization can be achieved through the presence of physiological concentrations of zinc and citrate (43). Thus, restoration of zinc levels and, thereby, prevention of low citrate concentrations, together, may prevent ZnR/GPR39 mediated enhancement of prostate tumor growth.

The decrease in zinc, along with the corresponding decrease in citrate levels, is the most consistent and persistent existing hallmark characteristic that differentiates prostate cancer from normal and benign prostate.

### The Prevalence of Low Zinc Status in Men Over 50 and Its Endogenous and Exogenous Causes: A Need for Zinc Supplementation?

In the light that more than half of men in their 60's suffer from BPH, which increases to 90% by 70–80 years of age, and the direct effects of zinc on normal or malignant prostate cells, the need of adequate zinc status in men for having a healthy prostate becomes evident. Prostate zinc levels are depending on circulating zinc concentrations that have to be considered (29), which puts a focus on maximizing cellular uptake of zinc in tissues and absorption of zinc in the intestines. It has been shown in several studies that older adults (older adults >50 years) frequently have low zinc status (44, 45). Although the daily requirement for zinc does not increase with age, lifestyle factors and a reduced capacity to absorb zinc, an increase in the likelihood of diseases that affect zinc utilization, and the use of drugs that may decrease the bioavailability of zinc may all contribute to putting older individuals at an increased risk for the development of a mild zinc deficiency (Table 1).

**Table 1:** Summary of endogenous and exogenous factors that may contribute to low zinc status in older men.

**Endogenous factors**		**Exogenous factors**	
**Morbidities that provoke Zn excretion**	**Morbidities that prevent Zn absorption**	**Diet**	**Drugs**
Severe or persistent diarrhea (46, 47)	Malabsorption syndromes (48), celiac disease (49–53), and short bowel syndrome (54)	Lack of Zn intake in the elderly due to restricted food choices and patterns (55–57)	Omeprazole and other Proton Pump Inhibitor medications (58–60)
Inflammatory bowel disease, including Crohn's disease and ulcerative colitis (61–65)	Gastrointestinal cancers	Presence of dietary factors that influence zinc absorption. E.g., Phytic acids (66–71)	Medications like tetracycline and quinolone antibiotics (72–74)
Alcoholic liver disease (75–79)			Metal-chelating agents, such as penicillamine, diethylenetriamine pentaacetate (DTPA) (80–83)
Chronic renal disease (84–87)			Anticonvulsant drugs (sodium valproate) (88–91)

Older adults that are vulnerable to the onset of BPH and PCa, therefore, may need zinc supplementation. Men with BPH and PCa have an increase in zinc excretion compared with healthy men (38); at the same time, some comorbidities are also known to provoke zinc excretion such as severe or persistent diarrhea, inflammatory bowel diseases, and renal disease. Recent studies and meta-analyses have found an association between inflammatory bowel disease and PCa (92–94). Besides, endogenous factors that prevent zinc absorption are more frequently present in older men, such as malabsorption syndromes (e.g., celiac disease and short bowel syndrome), and GI cancers.

Besides these endogenous factors, exogenous factors can lead to lower zinc status in older adults, such as limited food choices leading to a less variable diet that may include dietary factors that lower the bioavailability of zinc, or that are low in zinc content. For example, the evaluation of zinc intakes using dietary reference intakes, recommended dietary allowances, and estimated average requirements for elderly adults (60 years and older) showed that the prevalence of inadequate dietary intake zinc was 35–41% for males, and 36–45% for females (44).

The requirement for dietary zinc may be as much as 50% greater for individuals on a vegetarian diet whose major food staples are grains and legumes because high levels of phytate in these foods reduce zinc absorption (44, 95).

Phytic acid (PA) binds zinc in the gastrointestinal tract, thereby diminishing the bioavailability of zinc (66–68, 96). In addition, various other factors can influence zinc absorption. For example, drug interactions with zinc may decrease its absorption or provoke its excretion. A widely used drug is omeprazole, used for the treatment of acid reflux disease by decreasing stomach acid levels. According to a study, patients taking omeprazole showed a marked increase of zinc deficiency (from 16 to 50% within 2 months) upon starting treatment with the drug (58).

Besides proton-pump inhibitors, chelating drugs used to treat metal toxicity (like diethylenetriamine pentaacetate used in the treatment of iron overload and penicillamine prescribed to treat the copper overload in Wilson's disease) cause serious side effects in the form of severe zinc deficiency (80). Additionally, anticonvulsant drugs, such as sodium valproate and constant usage of diuretics, may negatively affect zinc levels in patients.

Few studies investigated the effects of zinc supplementation so far. For example, a study reported a protective role for zinc in BPH (97). In addition, the accumulation of zinc in prostate tissue depends on the activity of zinc transporters such as ZnT4 (SLC30A4) and ZIP4 (SLC39A4) that can be modulated by nutraceuticals such as daidzein. A combination of zinc, daidzein and isolase improved clinical symptoms and quality of life in patients with LUTS due to BPH (98, 99)

### Supplementing Zinc for Maintaining Adequate Zinc Levels in Older Men: Not All the Sources of Zinc Are Alike

Taken together, more than 50% of men over 60 suffer from BPH, and also have low zinc status. Thus, there is a strong case for establishing a program of zinc supplementation in men over 50 due to the endogenous and exogenous factors presented above. Several studies showed that the treatment of malignant prostate cells with zinc that will increase cellular zinc levels leads to the inhibition of cell proliferation, promotion of apoptosis, and inhibition of cell migration and invasion (28). However, there are many options available for supplementation, and choosing the correct supplement may be critical to achieving effects in prostate tissues.

Not all sources of zinc deliver the metal to the body for utilization in the same way. Ionic zinc (Zn^2+^) from dietary sources is taken up in the intestine by the ZIP family of zinc transporters, transporting zinc from the gut lumen into the intestinal enterocytes. From the enterocytes, zinc is further transported into the bloodstream by another family of zinc transporters, the ZnT transporter family (100). As described above, there are many antagonists and drug-interactions that can inhibit the uptake and utilization of ionic zinc. Therefore, inorganic zinc (Zn^2+^) sources may not be the best choice for older men. As an alternative, many forms of organic ligands for zinc are available for human use. Common ones are citrates, gluconates, glycinates, and picolinate. Careful consideration should be given to the viability of the chemistry. If the ligand dissociates due to the low pH in the stomach, the supplement will effectively behave like an inorganic metal supplement. If the ligand stays bound, but the complex is not absorbed well, the delivered zinc will not become bioavailable.

Our recent studies show that certain amino acids used as ligands are promising for delivering the metal (69). Amino acids, for example, from digested proteins, are taken up by at least four sodium-dependent amino acid transporters, and sodium-independent transporters, mediating the uptake of acidic, basic, and neutral amino acids. It was found that the metal bonded to the amino acids (Zn-AA) can be transported through the amino acid transporter and, therefore, may not be affected by the absence of plasma membrane localized ZIP1 transporter in the malignant PCa cells. We recommend the combination of zinc bound to glutamate (Glu) and lysine (Lys). This has been determined to be an advantageous combination for both uptake into the enterocyte as well as the passage into the circulation (69). The Lys and Glu, in combination, utilize different transporters. It has also been determined that metal amino acid complexes are not affected by common antagonists the same way inorganic supplements are (69). Furthermore, once in the circulation, amino acid complexes are excreted at a slower rate than other sources of minerals. Methionine (Met) is another viable option being an essential amino acid but mainly utilizing only one amino acid transporter. Methionine complexes have been patented in the early 1970's (Pat. No. US3941818A) and are still available for the use in humans.

The way of cellular uptake and the protection from sequestering by metallothionines, as well as slower excretion profiles may give organic zinc supplements such as ZnAAs an advantage that could be especially beneficial for older individuals. In particular, because older individuals may have restricted dietary habits with nutrients low in zinc or with high concentrations of uptake antagonists. In addition, medications that are taken and that limit zinc availability may be less disruptive for ZnAAs. Besides, metallic amino acid complexes have been used as human supplements and have a long history of safety and efficacy. They are frequently used as a mineral supplement for animals, where extensive research data shows their effectiveness and advantages over inorganic supplements.

## Conclusions

The decrease in zinc and, subsequently, citrate levels is the most characteristic hallmark of prostate cancer. The important role of zinc as a regulator of apoptosis in prostate cells makes the decrease a likely cause for rather than a consequence of PCa. Therefore, an adequate dietary intake of zinc is essential for older adults. The consequences of only mild zinc deficiency, such as impaired immune system function, are especially relevant to the maintenance of health and may be critical in the prevention of age-related diseases. Especially in the prostate, adequate zinc status will help to maintain health and physiological function and prevent prostate disease from developing or further progressing, thereby acting as a vital anti-BPH and anti-PCa agent. Thus, targeted zinc supplementation with supplements tailored to the needs of the elderly, i.e., ZnAAs, should be considered.

## Author Contributions

HV, GV, PS, and EB-J drafted the manuscript. AS and AG edited and finalized the manuscript. AG prepared the figures. All authors contributed to the article and approved the submitted version.

## Conflict of Interest

GV and PS were employed by Zinpro Corporation, HV by Vela Staines y Asociados SA de CV. The remaining authors declare that the research was conducted in the absence of any commercial or financial relationships that could be construed as a potential conflict of interest.
